# Supplementary material for: Evidence of complex contagion of information in social media: An experiment using Twitter bots
Source: PLoS One. 2017 Sep 22;12(9):e0184148. doi: 10.1371/journal.pone.0184148 (PMC5609738; doi:10.1371/journal.pone.0184148)
Supplement: S1 Appendix — Explains and evaluates the procedure used to efficiently generate unordered subsets. (PDF) [file pone.0184148.s001.pdf]

S1 Appendix: Efficiently Generating Unique Unordered Subsets

In the following, we outline an efficient method to compute the Poisson binomial distributions over κ for the complex contagion model. The probability distributions

$$P(\kappa|A) = \sum_{s\in S_\kappa} \prod_{i\in s} (1-(1-q)^{a_i}) \prod_{i\notin s} (1-q)^{a_i}, \quad (1)$$

We observe that while each element of an instance of

$$S_\kappa = \{s \subseteq \{1, 2, \ldots, |A|\}, |s| = \kappa\}, \quad (2)$$

the subsets of an activity list A of size k, give a contribution to $P(\kappa|A)$, the contribution depends only on the elements of the set, and not on their order. As the number of these subsets is a combinatorial expression that grows very quickly with $|A|$ and k, and as the majority of the elements in A were ones (as most of the bots only participated once in each intervention), we were able to complete the otherwise infeasible exact computation of $P(\kappa|A)$ by devising a method to generate unique unordered subsets from A and then multiply each unique subset with its multiplicity, which could in turn be computed from simple combinatorial expressions.

To illustrate our approach, we first explain a common way (as implemented in the itertools module in Python 2.7) of generating all possible subsets of a given length of a set. As an example, we use $A = [2, 1, 2, 1, 3, 1, 1, 4]$ and $k = 3$. Three pointers are then initialized to the three first values, and the last of those is set as the 'active' pointer. A series of steps is then repeated until none are possible:

- Attempt to move the active pointer one step to the right.
- If the pointer falls off the array or runs into another pointer, then set to active the pointer to the left of the current one and attempt again.
  - Terminate if we run out of pointers, i.e. if no pointers can be moved anymore.
- When move is successful, generate the set of the values pointed to, move all pointers to the right of the active pointer to the positions immediately following it, and reset the 'active' status to the rightmost pointer.

This then generates subsets like $(2, 1, 2)$, $(2, 1, 1)$, … $(2, 1, 4)$, $(2, 2, 1)$, $(2, 2, 3)$ etc. until $(1, 1, 4)$ where no more pointers are able to move and the procedure terminates.

This approach has the disadvantage of recounting subsets that are identical or are permutations of each other, such as $(2, 1, 2)$ and $(2, 2, 1)$ above. We remedy this by adding to the above algorithm a preprocessing step in which the input list A is sorted, and then defining a new list S in which the i'th element denotes the position the active pointer should be moved to given that it is pointing to $A[i]$ now, i.e. the value of $S[i]$ is the value of the first index i' at which $A[i'] > A[i]$. Reusing the example above to illustrate this,

$$S = [4, 4, 4, 4, 6, 6, 7, 8],$$
$$A = [1, 1, 1, 1, 2, 2, 3, 4],$$

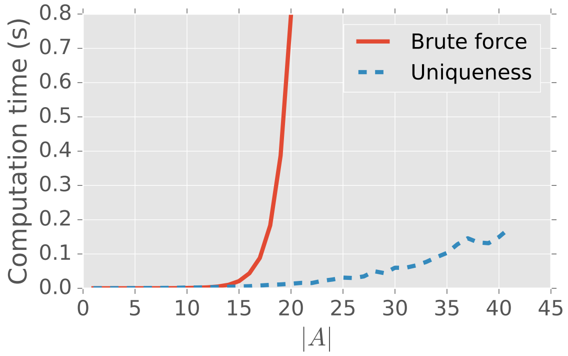

Figure 1: Time needed to compute $P(\kappa|A)$ using a brute force approach (solid red line) and our unique subset generation approach (dashed blue line). For each data point, we generated a simulated activity list with length $|A|$ with elements drawn with probabilities based on their frequency in our data, and computed $P(\kappa|A)$ using the two methods. The runtime is then averaged over 100 such computations in order to minimize noise. Our approach allows us to compute Poisson binomial distributions for activity lists of lengths that would otherwise be infeasible.

where the final index $i = 8$ falls off the array, consistent with the description above. This generates subsets like $(1, 1, 1)$, $(1, 1, 2)$, $(1, 1, 3)$, $\ldots$, $(1, 2, 2)$, $(1, 2, 3)$ etc. until $(2, 3, 4)$. The multiplicity of each such subset can be computed analytically, allowing one to compute the probability of drawing each subset.

When some elements of A occur very frequently, this approach, which we call the 'uniqueness' approach here as it only counts unique combinations, results in a clear improvement over the brute force approach. Indeed, for our data this approach turned out necessary to perform an otherwise infeasible computation. To illustrate this, we ran both method on simulated lists A of varying lengths, constructing each list A by randomly drawing elements from a distribution representative of our data. The resulting runtimes are shown in figure 1 and clearly show the speedup.
