# Supplementary material for: Evidence of complex contagion of information in social media: An experiment using Twitter bots
Source: PLoS One. 2017 Sep 22;12(9):e0184148. doi: 10.1371/journal.pone.0184148 (PMC5609738; doi:10.1371/journal.pone.0184148)
Supplement: S2 Appendix — Demonstrates the validity of a Gaussian approximation to the Poisson binomial distribution. (PDF) [file pone.0184148.s002.pdf]

S2 Appendix: Gaussian approximation of the Poisson Binomial

In the following, we detail why the Poisson binomial distribution consisting of n draws fr om separate Bernoulli distributions with individual probabilities $p_1, p_2, \ldots, p_n$, and accordingly, variances $\sigma_i^2 = p_i(1-p_i)$ approaches $\mathcal{N}(\mu, \sigma^2)$, where $\mu = \sum_i p_i$ and $\sigma^2 = \sum_i p_i(1-p_i)$ for large n. This closely follows the proof of the central limit theorem using characteristic functions, but we include the derivation in case some readers are unfamiliar with the proof.

The characteristic function (c.f.) of a random variable X is defined as

$$\varphi_X(t) = \langle e^{itX} \rangle = \sum_{n=0}^{\infty} i^n \frac{\langle X^n \rangle}{n!} t^n, \tag{1}$$

which results in the properties

$$\varphi_{X+Y}(t) = \varphi_X(t) \cdot \varphi_y(t), \tag{2}$$
$$\varphi_{cX}(t) = \varphi_X(ct). \tag{3}$$

Note that the c.f. for a Gaussian with zero mean, $\mathcal{N}(0, \sigma^2)$, is $\mathrm{e}^{-\sigma^2 t^2/2}$. Rather than considering directly the random variables X_i drawn from the Poisson binomial, we first subtract from each the mean success probability, i.e. we transform them like $X_i \to X_i - \overline{p}$. This subtraction ensures that $\langle X_i \rangle = 0$ and $\langle X_i^2 \rangle = \sigma_i^2$, which simplifies the proof We also divide by the square root of the number of draws, as this makes the convergence easier to show. When the derivation is done, we can simply obtain the distribution of the sum by substituting $\mu \to \mu + \sum_i p_i$ and $\sigma \to \sqrt{n}\sigma$.

Writing out the c.f. for these random variables using (2) and (3) gives

$$\varphi_{\sum_i X_i/\sqrt{n}}(t) = \prod_{i=1}^{n} \varphi_{X_i/\sqrt{n}}(t) = \prod_{i=1}^{n} \varphi_{X_i}(t/\sqrt{n}) \tag{4}$$

Using the properties following from the variables having zero mean, the expansion in (1) becomes

$$\varphi_X(t) = 1 - \frac{\sigma^2}{2} t^2 + \mathcal{O}(t^3), \tag{5}$$

so we get

$$\varphi_{\sum_i X_i/\sqrt{n}}(t) = \prod_{i=1}^{n} \left(1 - \frac{\sigma_i^2}{2n} t^2 + \mathcal{O}\left(t^3/\sqrt{n}^3\right)\right). \tag{6}$$

Using the expansion of the natural log, $\ln(1-x) = \sum_{n=1}^{\infty} (-1)^n \frac{x^n}{n} = -x + \mathcal{O}(x^2)$, this may be rewritten as

$$\varphi_{\sum_i X_i/\sqrt{n}}(t) = \mathrm{e}^{\ln\left(\prod_{i=1}^{n}\left(1 - \frac{\sigma_i^2}{2n} t^2 + \mathcal{O}\left(t^3/\sqrt{n}^3\right)\right)\right)}, \tag{7}$$
$$= \mathrm{e}^{\sum_{i=1}^{n}\left(-\frac{\sigma_i^2}{2n} t^2 + \mathcal{O}\left(t^3/\sqrt{n}^3\right)\right)} \tag{8}$$

Noting that $\sum_{i=1}^{n} \sigma_i^2 = n \cdot \overline{\sigma^2}$, meaning that when the moments of X are bounded, the c.f. approaches

$$\lim_{n\to\infty} \varphi_{\sum_i X_i/\sqrt{n}}(t) = \mathrm{e}^{-\overline{\sigma^2} t^2/2}, \tag{9}$$

which is the c.f. for $\mathcal{N}(0, \overline{\sigma^2})$, meaning that the distribution goes to $\frac{1}{\sqrt{2\pi\overline{\sigma^2}}}\mathrm{e}^{-x^2/2\overline{\sigma^2}}$ as previously described, the distribution for the sum of the original random variables can then be obtained by substituting $x \to x - \sum_i p_i$ and $\overline{\sigma^2} \to n \cdot \overline{\sigma^2} = \sum_i p_i(1 - p_i)$.
