# Supplementary material for: Evidence of complex contagion of information in social media: An experiment using Twitter bots
Source: PLoS One. 2017 Sep 22;12(9):e0184148. doi: 10.1371/journal.pone.0184148 (PMC5609738; doi:10.1371/journal.pone.0184148)
Supplement: S3 Appendix — Describes the procedure for recording the data. (PDF) [file pone.0184148.s003.pdf]

S3 Appendix: Note on Data Collection

The bots were hosted on Amazon EC2 instances, ensuring uninterrupted operation. Each team collected the data about their bot's activity as well as the development of its Twitter network. A script installed by each team facilitated the data collection effort. Once per hour it queried the Twitter API for the list of friends and followers of the team's bot, as well as the content it recently (re)tweeted. The script would then send the data to the central server at DTU for storage and further analysis. By relegating the collection effort to each team we avoided the Twitter API call quotas, which would curb the process if we had used a single account to gather the data.

During the intervention period, another script, installed at the central server, used the Twitter Streaming API to collect tweets with predefined hashtags coming from all Twitter users. We combine the information collected by the teams with the record of all interventions-related tweets to analyze the dynamics of information spread.
